# Association of Major Depressive Symptoms With Endorsement of COVID-19 Vaccine Misinformation Among US Adults

**DOI:** 10.1001/jamanetworkopen.2021.45697

**Published:** 2022-01-21

**Authors:** Roy H. Perlis, Katherine Ognyanova, Mauricio Santillana, Jennifer Lin, James Druckman, David Lazer, Jon Green, Matthew Simonson, Matthew A. Baum, John Della Volpe

**Affiliations:** 1 Massachusetts General Hospital, Boston; 2 Harvard Medical School, Boston, Massachusetts; 3 Rutgers University, New Brunswick, New Jersey; 4 Boston Children’s Hospital, Boston, Massachusetts; 5 Northwestern University, Evanston, Illinois; 6 Northeastern University, Boston, Massachusetts; 7 Harvard University, Cambridge, Massachusetts

## Abstract

**Importance:**

Misinformation about COVID-19 vaccination may contribute substantially to vaccine hesitancy and resistance.

**Objective:**

To determine if depressive symptoms are associated with greater likelihood of believing vaccine-related misinformation.

**Design, Setting, and Participants:**

This survey study analyzed responses from 2 waves of a 50-state nonprobability internet survey conducted between May and July 2021, in which depressive symptoms were measured using the Patient Health Questionnaire 9-item (PHQ-9). Survey respondents were aged 18 and older. Population-reweighted multiple logistic regression was used to examine the association between moderate or greater depressive symptoms and endorsement of at least 1 item of vaccine misinformation, adjusted for sociodemographic features. The association between depressive symptoms in May and June, and new support for misinformation in the following wave was also examined.

**Exposures:**

Depressive symptoms.

**Main Outcomes and Measures:**

The main outcome was endorsing any of 4 common vaccine-related statements of misinformation.

**Results:**

Among 15 464 survey respondents (9834 [63.6%] women and 5630 [36.4%] men; 722 Asian respondents [4.7%], 1494 Black respondents [9.7%], 1015 Hispanic respondents [6.6%], and 11 863 White respondents [76.7%]; mean [SD] age, 47.9 [17.5] years), 4164 respondents (26.9%) identified moderate or greater depressive symptoms on the PHQ-9, and 2964 respondents (19.2%) endorsed at least 1 vaccine-related statement of misinformation. Presence of depression was associated with increased likelihood of endorsing misinformation (crude odds ratio [OR], 2.33; 95% CI, 2.09-2.61; adjusted OR, 2.15; 95% CI, 1.91-2.43). Respondents endorsing at least 1 misinformation item were significantly less likely to be vaccinated (crude OR, 0.40; 95% CI, 0.36-0.45; adjusted OR, 0.45; 95% CI, 0.40-0.51) and more likely to report vaccine resistance (crude OR, 2.54; 95% CI, 2.21-2.91; adjusted OR, 2.68; 95% CI, 2.89-3.13). Among 2809 respondents who answered a subsequent survey in July, presence of depression in the first survey was associated with greater likelihood of endorsing more misinformation compared with the prior survey (crude OR, 1.98; 95% CI, 1.42-2.75; adjusted OR, 1.63; 95% CI, 1.14-2.33).

**Conclusions and Relevance:**

This survey study found that individuals with moderate or greater depressive symptoms were more likely to endorse vaccine-related misinformation, cross-sectionally and at a subsequent survey wave. While this study design cannot address causation, the association between depression and spread and impact of misinformation merits further investigation.

## Key Points

**Question:**

Are major depressive symptoms associated with increased risk of believing common misinformation about COVID-19 vaccines among US adults?

**Findings:**

In this survey study including 15 464 US adults, people with moderate or greater major depressive symptoms on an initial survey were more likely to endorse at least 1 of 4 false statements about COVID-19 vaccines on a subsequent survey, and those who endorsed these statements were half as likely to be vaccinated.

**Meaning:**

These findings suggest another potential benefit of public health efforts to address depressive symptoms, namely reducing susceptibility to misinformation.

## Teaser

This survey study examines the association of moderate or greater depressive symptoms with believing COVID-19 vaccine–related misinformation.

## Introduction

The potential for misinformation to impact public health behavior was recognized prior to the COVID-19 pandemic,^1^ but since the onset of the pandemic, the consequences of misinformation have become even more apparent. Popular misperceptions are associated with hindering efforts to mitigate the spread and consequences of the SARS-CoV-2 virus by minimizing perceived risk of infection, discouraging masking and distancing behaviors, and reducing vaccination rates.^2,3^

While misinformation is increasingly well studied, most of this work has concentrated on how and why such misinformation spreads. Less understood are individual characteristics, beyond simple demographics and political affiliation, associated with greater susceptibility to misinformation, such as examined in a study by Druckman et al.^4^ Notably, misleading news stories inspiring negative emotions, such as disgust, have been found to spread more rapidly on social media.^5^ A general bias toward negativity in information selection, processing, and recall^6,7^ may exacerbate misinformation exposure. In the context of political misinformation, both anger and anxiety are associated with promoting beliefs in certain types of false stories.^8^

During the COVID-19 pandemic, approximately one quarter of adults in the US have consistently endorsed moderate or greater depressive symptoms.^9,10^ As depressive symptoms may contribute to negativity bias, we hypothesized that such symptoms would be associated with greater receptivity to misinformation, with potentially profound associations with health-related behaviors.

We used data from a 50-state US survey to examine this hypothesized association in 2 ways. First, with cross-sectional data from more than 15 000 individuals, we characterized the association between presence of depressive symptoms and endorsement of misinformation. Second, examining the subset of individuals who completed 2 waves of the survey approximately 1 month apart, we examined the extent to which depressive symptoms on the initial survey were associated with endorsement of new misinformation 1 month later. We then examined potential mediators or moderators of these associations and the association between misinformation and vaccination status.

## Method

This survey study was reviewed by the institutional review board of Harvard University and determined to be exempt; all participants signed informed consent online prior to survey access. In reporting results, we follow the American Association for Public Opinion Research (AAPOR) reporting guideline for survey studies.

### Study Design

The COVID States Project^11^ survey has been conducted approximately once every 6 weeks since April 2020. Of note, participants are not aware that they are completing a survey focused on COVID-19 a priori, in an effort to limit selection bias. Our analysis used the 2 waves conducted between April 1 and May 3, 2021, and between June 9 and July 7, 2021, which included questions about vaccine-related misinformation. This online survey applies nonprobability sampling and representative quotas to balance age, gender, and race and ethnicity across 50 states and the District of Columbia. That is, instead of randomly sampling the full US population as in probability sampling (eg, by random digit dialing), for reasons of feasibility, this survey samples individuals who choose to participate in online surveys, but applies quotas and reweighting to approximate the US adult population in each state. Each adult in the population thus does not have an equivalent probability of being selected. Survey results were weighted based on US Census data to balance on age, gender, race and ethnicity, education, region, and rural or urban area of residence.

### Measures

We assessed vaccine-related misinformation using 4 statements, which respondents were asked to rate as accurate (statement is true), inaccurate (statement is not true), or not sure. We selected these statements based on misinformation prevalent on social media platforms in spring 2021. Specific statements of misinformation included “The COVID-19 vaccines will alter people’s DNA,” “The COVID-19 vaccines contain microchips that could track people,” “The COVID-19 vaccines contain the lung tissue of aborted fetuses,” and “The COVID-19 vaccines can cause infertility, making it more difficult to get pregnant.” At the conclusion of this survey section, all respondents were informed which items were not true, to ensure that the survey itself did not facilitate spread of misinformation. For cross-sectional analysis, as in our prior work,^4^ we categorized any accurate responses as reflecting belief in misinformation. For longitudinal analysis, we categorized an increase in the number of statements labeled accurate as worsening belief in misinformation, for example, going from no statements labeled accurate in the first wave to 1 or more statements labeled accurate in the second wave.

Survey participants also completed the Patient Health Questionnaire 9-item (PHQ-9) as a measure of major depressive symptoms over the preceding 2 weeks.^12^ In primary care settings, a value of 10 or greater represents at least moderate depression and is often applied as the threshold for treatment; therefore, we elected a priori to examine presence or absence of major depressive symptoms at this threshold, as in our prior work using these survey items, rather than assuming a linear or dose-response association between depression and misinformation.

Additional survey items asked respondents whether they used particular social media platforms and whether they had used any of a list of news sources (including MSNBC, Fox News, CNN, Newsmax, Facebook, and the Biden administration) as sources of COVID-19–related news over the prior 24 hours. Sociodemographic features, including race, ethnicity, and gender, were identified by self-report. Race and ethnicity data were collected to ensure representativeness of the US population for the survey as a whole. Region (ie, Northeast, South, Midwest, and West) and urban or rural status were assigned based on zip code. Ideology was assessed using a 7-point scale (range, 1 to 7, with 1 indicating extremely liberal; 4, moderate; and 7, extremely conservative). Political party affiliation was determined by asking, “Generally speaking, do you think of yourself as a…” with Democrat, Republican, Independent, and other as options; for analytic purposes other and Independent were combined in a single category. Respondents were also asked if they had received at least 1 COVID-19 vaccination; if they had not, they were further asked “If you were able to choose when to get a COVID-19 vaccine, would you get it…”, with response options including “as soon as possible,” “after at least some people I know,” “after most people I know,” or “I would not get the COVID-19 vaccine.” The last category was considered to be vaccine resistant.

### Statistical Analysis

For purposes of primary analysis, we applied multiple logistic regression to examine the association between presence of at least moderate depressive symptoms by PHQ-9 and endorsing at least 1 item of vaccine-related misinformation. These models were fit without adjustment and then with adjustment for sociodemographic features, including age, gender, race and ethnicity (captured using US Census categories), level of education, urban, suburban, or rural location, and region. Survey results were reweighted using interlocking national weights for age, gender, and race and ethnicity, education, and region, applying the survey package in R statistical software version 4.0 (R Project for Statistical Computing).

We also examined the possibility that individuals with depressive symptoms might be less confident in their responses to questions about misinformation, as indicated by presence of at least 1 not sure answer to misinformation questions. These analyses used logistic regression models, with the same covariates used to examine presence of misinformation.

In secondary analysis, we examined potential mediating or moderating associations of social media use, news sources, and trust in institutions with the association between mood and misinformation. That is, we considered the possibility that these associations could arise in association with mood and media use, news consumption, or willingness to trust institutions. We analyzed social media use via terms for self-reported use of Twitter, Facebook, TikTok, and Instagram, and news sources for receiving COVID-19 information within the past 24 hours, including CNN, MSNBC, Fox News, Newsmax, Facebook, or the Biden administration. We then examined self-reported trust in institutions (ie, the White House, the Food and Drug Administration, and the Centers for Disease Control and Prevention), in hospitals and physicians, in scientists, and in news media. For each of the trust variables, we asked, “How much do you trust the following people and organizations to do the right thing to best handle the current coronavirus (COVID-19) outbreak?” and used a 4-point scale from 1 indicating not at all to 4, a lot. To understand the association between misinformation and vaccine-related behavior, we compared rates of vaccination and rates of vaccine resistance among individuals who did or did not endorse misinformation.

Finally, we used the subset of individuals who responded to both the April to May and June to July waves to analyze whether presence of depression in April to May was associated with incident (rather than prevalent) misinformation (ie, emergence of additional misinformation from one wave to the next). We again used multiple logistic regression to adjust for sociodemographic features, with national reweighting, using noninterlocking weights. *P* values were 2-sided, and statistical significance was set at *P* < .05.

## Results

Among 15 464 survey respondents (9834 [63.6%] women and 5630 [36.4%] men; mean [SD] age, 47.9 [17.5] years), 722 respondents (4.7%) identified as Asian, 1494 respondents (9.7%) identified as Black, 1015 respondents (6.6%) identified as Hispanic, and 11 863 respondents (76.7%) identified as White. A total of 4164 respondents (26.9%) had moderate or greater depressive symptoms on the PHQ-9 (Table 1), and 2964 respondents (19.2%) endorsed at least 1 vaccine-related statement of misinformation (Table 1).

**Table 1.** Characteristics of Initial Survey Cohort

Characteristic	Respondents, No. (%)			*P* value
	PHQ-9 score		Full cohort total (N = 15 464)	
	<10 (n = 11 300)	≥10 (n = 4164)		
Age, mean (SD), y	50.88 (17.14)	39.72 (15.75)	47.88 (17.49)	<.001
Gender				
Women	7155 (63.3)	2679 (64.3)	9834 (63.6)	.24
Men	4145 (27.7)	1485 (35.7)	5630 (36.4)	
≥College education	5312 (47.0)	1643 (39.5)	6955 (45.0)	<.001
Race and ethnicity				
Asian	490 (4.3)	232 (5.6)	722 (4.7)	<.001
Black	1117 (9.9)	377 (9.1)	1494 (9.7)	
Hispanic	660 (5.8)	355 (8.5)	1015 (6.6)	
White	8797 (77.8)	3066 (73.6)	11 863 (76.7)	
Other^a^	236 (2.1)	134 (3.2)	370 (2.4)	
Region				
Midwest	2891 (25.6)	981 (23.6)	3872 (25.0)	<.001
Northeast	1928 (17.1)	625 (15.0)	2553 (16.5)	
South	4284 (37.9)	1678 (40.3)	5962 (38.6)	
West	2197 (19.4)	880 (21.1)	3077 (19.9)	
Urbanicity				
Rural	1698 (15.0)	589 (14.1)	2287 (14.8)	<.001
Suburban	6665 (59.0)	2342 (56.2)	9007 (58.2)	
Urban	2937 (26.0)	1233 (29.6)	4170 (27.0)	
Employed	5991 (53.0)	2439 (58.6)	8430 (54.5)	<.001
Ideology score. mean (SD)^b^	4.03 (1.68)	3.45 (1.72)	3.87 (1.71)	<.001
Political party				
Republican	3227 (28.6)	867 (20.8)	4094 (26.5)	<.001
Democratic	4523 (40.0)	1954 (46.9)	6477 (41.9)	
Independent	3550 (31.4)	1343 (32.3)	4893 (31.6)	
PHQ-9 score, mean (SD)	3.13 (2.95)	16.05 (4.87)	6.61 (6.76)	<.001
Social media use				
Facebook	8721 (77.2)	3153 (75.7)	11 874 (76.8)	.06
Instagram	5037 (44.6)	2473 (59.4)	7510 (48.6)	<.001
TikTok	2205 (19.5)	1514 (36.4)	3719 (24.0)	<.001
Twitter	3539 (31.3)	1778 (42.7)	5317 (34.4)	<.001
Trust in institutions^c^				
White House	3199 (28.4)	1059 (25.5)	4258 (27.6)	<.001
FDA	3506 (31.1)	1249 (30.0)	4755 (30.8)	.21
CDC	4687 (41.6)	1713 (41.3)	6400 (41.5)	.77
Hospitals and doctors	7456 (66.1)	2501 (60.3)	9957 (64.5)	<.001
Scientists and researchers	6067 (53.8)	2138 (51.6)	8205 (53.2)	.01
News media companies	1133 (10.1)	572 (13.8)	1705 (11.1)	<.001
Social media companies	710 (6.3)	503 (12.2)	1213 (7.9)	<.001
News sources for COVID-19				
CNN	3549 (31.4)	1724 (41.4)	5273 (34.1)	<.001
Fox News	3287 (29.1)	1413 (33.9)	4700 (30.4)	<.001
MSNBC	1613 (14.3)	660 (15.9)	2273 (14.7)	.01
Biden administration	2407 (21.3)	870 (20.9)	3277 (21.2)	.58
Facebook	3350 (29.6)	1658 (39.8)	5008 (32.4)	<.001
Newsmax	525 (4.6)	183 (4.4)	708 (4.6)	.51
Misinformation statements endorsed				
Mean (SD), No.	0.27 (0.76)	0.62 (1.11)	0.37 (0.88)	<.001
≥1 Statement endorsed	1715 (15.2)	1249 (30.0)	2964 (19.2)	<.001
Vaccinated	6095 (53.9)	1828 (43.9)	7923 (51.2)	<.001

Abbreviations: CDC, Centers for Disease Control and Prevention; FDA, Food and Drug Administration; PHQ-9, Patient Health Questionnaire 9-item.

^a^ Other race and ethnicity includes Native American or Alaska Native and Pacific Islander or Native Hawaiian.

^b^ Ideology was assessed using a 7-point scale ranging from 1 to 7, with 1 indicating extremely liberal and 7, extremely conservative.

^c^ Given as the number and percentage of respondents who reported feeling a lot of trust in the institution. Institutional trust data were incomplete for 34 respondents regarding White House, 29 respondents regarding the FDA, 35 respondents regarding the CDC, 32 respondents regarding hospitals and physicians, 48 respondents regarding scientists and researchers, 49 respondents regarding news media, and 87 respondents regarding social media companies.

**Figure 1.**
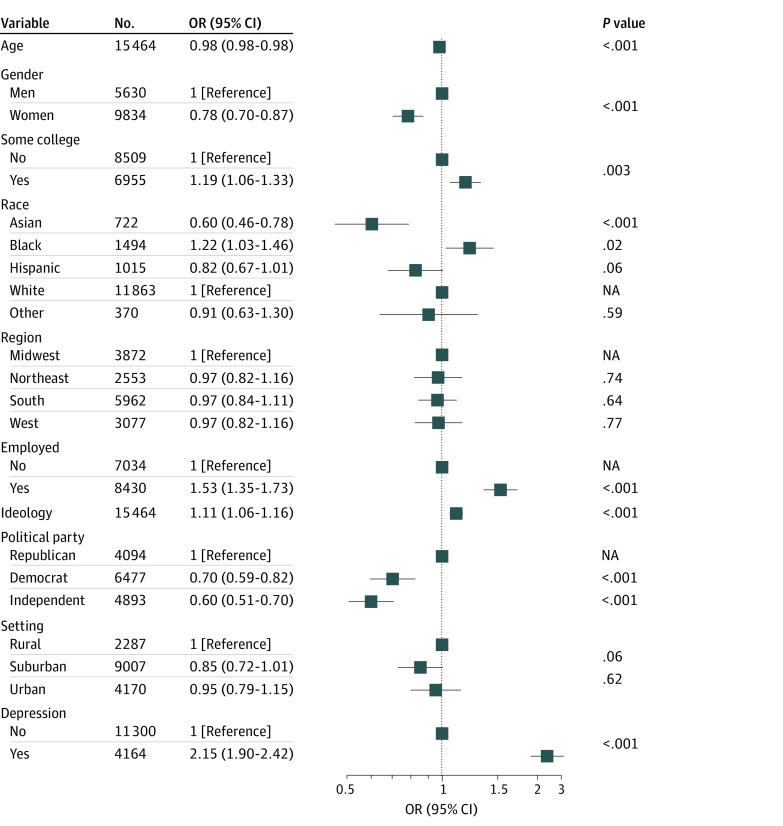
Regression Model for Endorsing at Least 1 Misinformation Item in Initial Survey Wave Other race includes Native American or Alaska Native and Pacific Islander or Native Hawaiian. Ideology was assessed using a 7-point scale, (range, 1-7, with 1 indicating extremely liberal and 7, extremely conservative). The odds ratio (OR) was calculated per 1-point increase. NA indicates not applicable.

In a reweighted analysis to reflect the US population as a whole, 29.3% (SE, 0.9%) of respondents with moderate or greater depression endorsed misinformation, compared with 15.1% (SE, 0.4%) of those without. Presence of depression was significantly associated with increased likelihood of endorsing misinformation (crude odds ratio [OR], 2.33; 95% CI, 2.09-2.61; adjusted OR, 2.15; 95% CI, 1.91-2.43) (Figure 1). Individuals with moderate depression were also more likely to indicate that they were not sure about at least 1 item of misinformation, although the association was no longer significant after adjustment (crude OR, 1.25; 95% CI, 1.14-1.38; adjusted OR, 1.10; 95% CI, 0.99-1.22).

We next examined potential factors associated with mediating or moderating this association by considering whether the association between misinformation and depression was meaningfully changed by addition of terms to the multiple regression models. Table 2 shows the base model and the adjusted ORs associating misinformation with depression in models incorporating social media, news source, or trust variables. While all of these variables were significantly associated with misinformation, in all additional models, the ORs changed by less than 10%, suggesting modest mediating or moderating associations at best.

**Table 2.** Associations Between Presence of Depression and Presence of Misinformation, With Incorporation of Potential Mediators or Moderators

Factor	OR (95% CI)^a^
Base model	2.15 (1.90-2.42)
Institutional trust	
White House	2.10 (1.86-2.38)
FDA	2.10 (1.86-2.37)
CDC	2.12 (1.87-2.40)
Hospitals and physicians	2.04 (1.80-2.31)
Scientists	2.02 (1.78-2.29)
News media	2.15 (1.91-2.43)
Social media	2.14 (1.90-2.41)
Social media platforms	
Facebook	2.15 (1.90-2.42)
Instagram	2.15 (1.90-2.42)
TikTok	2.14 (1.90-2.42)
Twitter	2.14 (1.90-2.42)
Snapchat	2.14 (1.90-2.41)
YouTube	2.15 (1.91-2.43)
COVID-19 news sources	
CNN	2.13 (1.89-2.40)
Fox News	2.08 (1.84-2.34)
MSNBC	2.15 (1.90-2.42)
Biden administration	2.15 (1.91-2.43)
Facebook	2.12 (1.88-2.40)
Newsmax	2.12 (1.88-2.39)

Abbreviations: CDC, Centers for Disease Control and Prevention; FDA, Food and Drug Administration; OR, odds ratio.

^a^ In all analyses, *P* < .001.

To understand the potential real-world correlates of misinformation, we then examined vaccination status. Respondents endorsing at least 1 misinformation item were significantly less likely to be vaccinated (crude OR, 0.40; 95% CI, 0.36-0.45; adjusted OR, 0.45; 95% CI, 0.40-0.51) and significantly less likely to report a vaccinated family member (crude OR, 0.50; 95% CI, 0.45-0.56; adjusted OR, 0.55; 95% CI, 0.49-0.62). Among individuals who had not yet been vaccinated, those endorsing at least 1 misinformation item were more likely to report vaccine resistance (crude OR, 2.54; 95% CI, 2.21-2.91; adjusted OR, 2.68; 95% CI, 2.89-3.13).

**Figure 2.**
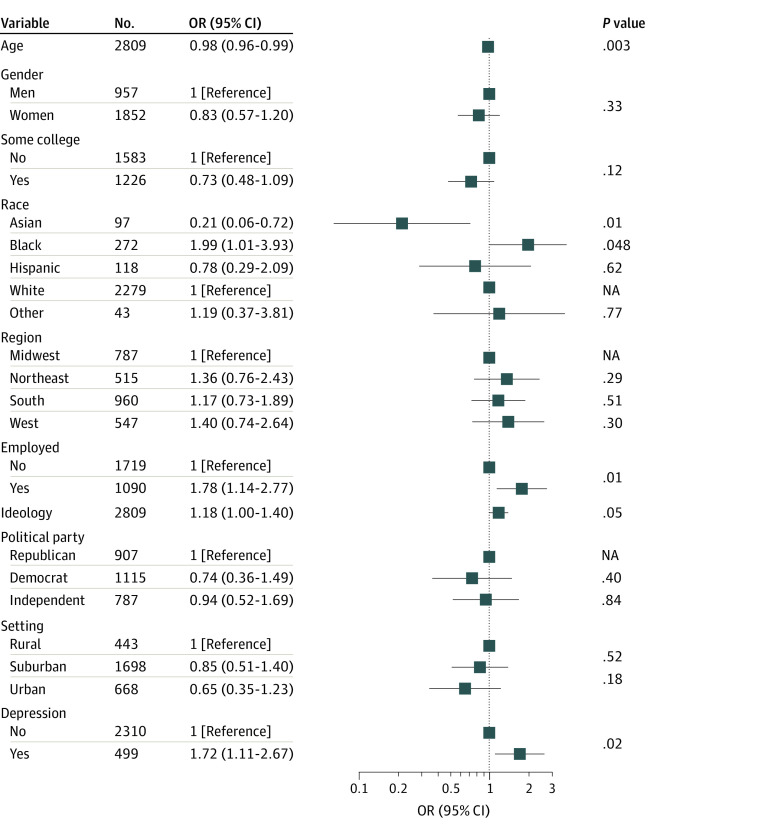
Regression Model of Worsening of Misinformation in Subsequent Survey, Based on Initial Survey Other race includes Native American or Alaska Native and Pacific Islander or Native Hawaiian. Ideology was assessed using a 7-point scale, (range, 1-7, with 1 indicating extremely liberal and 7, extremely conservative). The odds ratio (OR) was calculated per 1-point increase. NA indicates not applicable.

Finally, we considered 2809 individuals who answered a subsequent survey in June and July as a means of analyzing risk of incident misinformation. Characteristics of those individuals are summarized in Table 3; they included 1852 (65.9%) women and 957 (34.2%) men. A total of 97 respondents (3.5%) were Asian, 272 respondents (9.7%) were Black, 118 respondents (4.2%) were Hispanic, and 2279 respondents (81.1%) were White. The mean (SD) age was 58.1 (15.3) years. A total of 499 respondents (17.8%) were at least moderately depressed by PHQ-9 score in the April and May wave, and 370 respondents (13.2%) endorsed at least 1 item of misinformation. Presence of depression in the first survey was associated with greater likelihood of reporting more misinformation (that is, an increase in the number of items of misinformation rated as accurate) compared with the prior survey (crude OR, 2.03; 95% CI, 1.33-3.09; adjusted OR, 1.72; 95% CI, 1.11-2.67) (Figure 2). When terms for use of social media platforms were added, associations remained statistically significant (adjusted OR, 1.68; 95% CI, 1.08-2.61).

**Table 3.** Characteristics of Respondents Who Returned for a Subsequent Survey

Characteristic	Respondents, No. (%)			*P* value
	PHQ-9		Full cohort (n = 2809)	
	<10 (n = 2310)	≥10 (n = 499)		
Age, mean (SD), y	60.08 (14.35)	48.79 (16.03)	58.08 (15.28)	<.001
Gender				
Women	1500 (64.9)	352 (70.5)	1852 (65.9)	.02
Men	810 (35.1)	147 (29.5)	957 (34.1)	
≥College education	1026 (44.4)	200 (40.1)	1226 (43.6)	.08
Race and ethnicity				
Asian	78 (3.4)	19 (3.8)	97 (3.5)	.01
Black	227 (9.8)	45 (9.0)	272 (9.7)	
Hispanic	83 (3.6)	35 (7.0)	118 (4.2)	
White	1887 (81.7)	392 (78.6)	2279 (81.1)	
Other^a^	35 (1.5)	8 (1.6)	43 (1.5)	
Region				
Midwest	636 (27.5)	151 (30.3)	787 (28.0)	.46
Northeast	434 (18.8)	81 (16.2)	515 (18.3)	
South	789 (34.2)	171 (34.3)	960 (34.2)	
West	451 (19.5)	96 (19.2)	547 (19.5)	
Urbanicity				
Rural	368 (15.9)	75 (15.0)	443 (15.8)	.75
Suburban	1389 (60.1)	309 (61.9)	1698 (60.4)	
Urban	553 (23.9)	115 (23.0)	668 (23.8)	
Employed	852 (36.9)	238 (47.7)	1090 (38.8)	<.001
Ideology, mean (SD)^b^	4.24 (1.71)	3.79 (1.82)	4.16 (1.74)	<.001
Political party				
Republican	759 (32.9)	148 (29.7)	907 (32.3)	.002
Democratic	882 (38.2)	233 (46.7)	1115 (39.7)	
Independent	669 (29.0)	118 (23.6)	787 (28.0)	
PHQ-9 score, mean (SD)	2.51 (2.79)	15.70 (4.98)	4.85 (6.02)	<.001
Social media use				
Facebook	1763 (76.3)	391 (78.4)	2154 (76.7)	.33
Instagram	725 (31.4)	227 (45.5)	952 (33.9)	<.001
TikTok	189 (8.2)	104 (20.8)	293 (10.4)	<.001
Twitter	525 (22.7)	170 (34.1)	695 (24.7)	<.001
Trust in institutions^c^				
White House	655 (28.4)	140 (28.1)	795 (28.3)	.90
FDA	725 (31.4)	143 (28.7)	868 (30.9)	.23
CDC	932 (40.4)	206 (41.4)	1138 (40.6)	.69
Hospitals and physicians	1550 (67.2)	297 (59.8)	1847 (65.8)	.002
Scientists and researchers	1222 (53.0)	239 (48.1)	1461 (52.1)	.046
News media companies	238 (10.3)	71 (14.2)	309 (11.0)	.01
Social media companies	108 (4.7)	41 (8.2)	149 (5.3)	.001
News sources for COVID-19				
CNN	601 (26.0)	171 (34.3)	772 (27.5)	<.001
Fox News	633 (27.4)	157 (31.5)	790 (28.1)	.07
MSNBC	327 (14.2)	77 (15.4)	404 (14.4)	.46
Biden Administration	530 (22.9)	115 (23.0)	645 (23.0)	.96
Facebook	488 (21.1)	155 (31.1)	643 (22.9)	<.001
Newsmax	127 (5.5)	19 (3.8)	146 (5.2)	.12
Misinformation statements endorsed				
Mean (SD), No.	0.20 (0.68)	0.43 (0.94)	0.24 (0.74)	<.001
Increase in misinformation statements endorsed	136 (5.9)	55 (11.0)	191 (6.8)	<.001
Vaccinated	1414 (61.2)	258 (51.7)	1672 (59.5)	<.001

Abbreviations: CDC, Centers for Disease Control and Prevention; FDA, Food and Drug Administration; PHQ-9, Patient Health Questionnaire 9-item.

^a^ Other race and ethnicity includes Native American or Alaska Native and Pacific Islander or Native Hawaiian.

^b^ Ideology was assessed using a 7-point scale ranging from 1 to 7, with 1 indicating extremely liberal and 7, extremely conservative.

^c^ Given as the number and percentage of respondents who reported feeling a lot of trust in the institution. Institutional trust data incomplete for 4 respondents regarding the White House, 2 respondents regarding the FDA, 3 respondents regarding the CDC, 4 respondents regarding hospitals and doctors, 7 respondents regarding scientists and researchers, 6 respondents regarding news media, and 10 respondents regarding social media companies.

## Discussion

In this survey study using national data including more than 15 000 respondents, we found that presence of moderate or greater depressive symptoms was associated with greater likelihood of endorsing misinformation about vaccines, an association that persisted with adjustment for sociodemographic features as well as self-reported ideology and political party affiliation. This cross-sectional study design does not allow us to investigate causation, so the nature of the association between these features remains to be determined. However, using a subset of participants from the first wave who returned for the second, we found that depressive symptoms preceded misinformation emergence, suggesting that misinformation was unlikely to cause depression per se.

In general, negative biases are apparent in information processing even in the absence of depression.^6,7^ Individuals with major depressive symptoms often exhibit a more pronounced negativity bias, a form of attentional bias in which thoughts with negative valence receive greater focus.^13^ Insofar as forms of misinformation that elicit negative affect may be more likely to spread,^5^ it follows that depression could facilitate uptake of misinformation at an individual level.

Alternatively, it is possible that the association between depression and misinformation could be mediated by change in trust. Individuals with depression could exhibit less willingness to trust institutions attempting to combat misinformation, such as the Centers for Disease Control and Prevention, or greater willingness to trust other institutions that distribute misinformation. However, we found that incorporating terms for trust in these institutions in regression models did not change the main association with depression, which does not support a mediating association of trust in institutions.

As anticipated, we also found that individuals who embraced health misinformation were less likely to be vaccinated or be willing to get the vaccine if available. As such, individuals already burdened with depression may be at a higher risk of COVID-19. While beyond the scope of the present work, it bears noting that individuals with depression may also exhibit a lack of positive interpretation bias,^14^ ie, less optimistic beliefs,^15^ which could lead them to underestimate the potential benefit of vaccination. Notably, mood disorders have been associated with worse COVID-19 outcomes among hospitalized patients.^16^

### Limitations

This study has some limitations. While we adjusted for a range of sociodemographic features, we cannot exclude the role of confounding in the observed association. One potential confounder could be the use of social media: it is possible that more individuals with depression are more prone to use certain forms of social media, and those platforms may be more likely to promote misinformation. Alternatively, social media use could promote both depression^17,18,19^ and misinformation^1^ independently. Similarly, depression might be associated with different choices in news media. However, adding terms for individual social media platforms or news sources to regression models did not substantially change the associations between depression and misinformation, suggesting this is less likely to be the case.

## Conclusions

This survey study found that individuals with moderate or greater depressive symptoms were more likely to endorse vaccine-related misinformation, cross-sectionally and at a subsequent survey wave. While associative by necessity, our results more broadly suggest the importance of directly testing causation in future experiments, for example, by manipulating negativity bias and measuring the receptivity to misinformation. If causation could be established, it might suggest strategies aimed at reducing the consequences of depression in terms of misinformation. To date, efforts to combat the impact of misinformation on public health predominantly emphasize reduction in supply. In parallel, it may be possible to develop interventions targeting negativity bias that reduce demand, or at least modulate the capacity of misinformation to impact health decision-making.
